# Biological Control of Date Palm Mite *Oligonychus afrasiaticus* (McGregor) (Acari: Tetranychidae) Using the Predatory Mite *Euseius scutalis* (Athias-Henriot) (Acari: Phytoseiidae) Under Laboratory and Field Conditions

**DOI:** 10.3390/biology15181651

**Published:** 2026-09-18

**Authors:** Mahmoud M. Al-Azzazy, Saleh S. Alhewairini

**Affiliations:** Department of Plant Protection, College of Agriculture and Food, Qassim University, P.O. Box 6622, Buraydah 51452, Saudi Arabia

**Keywords:** date palm orchards, release rate, life table parameters

## Abstract

The date palm mite *Oligonychus afrasiaticus* is one of the most destructive phytophagous pests in date palm orchards and can cause severe economic losses when not effectively managed. Although chemical acaricides remain the primary control method, their adverse effects on natural enemies, environmental quality, and human health underscore the need to reduce reliance on chemical inputs and develop sustainable biological control strategies, including the use of acarine predators. Accordingly, this study aimed to evaluate the efficacy of the predatory mite *Euseius scutalis* as a biocontrol agent against *O. afrasiaticus* under both laboratory and field conditions. In addition, the biological characteristics of *E. scutalis*, a key predator of *O. afrasiaticus*, were examined under controlled laboratory conditions. All experiments were carried out on kidney bean discs in an incubator at 32 ± 1 °C, 50% RH, with 14:10 h L:D. *Euseius scutalis* completed development, survival, and oviposition on *O. afrasiaticus* as prey. The average developmental period for males was 5.17 days, and for females, it was 5.64 days; the average adult female longevity was 11.67 days. The average daily and total egg production were 2.81 and 48.90 eggs, respectively. The sex ratio was 0.70 females, and the death rate of immature stages was 0.66%. The intrinsic rate of increase (*r_m_*) was 0.281 females/female/day, the mean generation time (*T_o_*) was 12.36 days, and the net reproduction rate (*R_o_*) was 14.25 females/female. Experimental releases of the predatory mite *E. scutalis* were conducted in conventional date palm (*Phoenix dactylifera* L.) orchards in the Qassim region of Saudi Arabia. Three predator release rates (200, 400, and 600 individuals per date palm tree) were evaluated on two date palm cultivars, Barhi and Sokary, to assess their effectiveness against *O. afrasiaticus*. The release rate of 200 individuals/date palm tree did not provide effective control of *O. afrasiaticus*. When it was released at 400 and 600 individuals per tree, *E. scutalis* maintained the *O. afrasiaticus* population below the economic threshold of 4 moving stages per fruit. Hence, it can be recommended, considering these findings, to increase the number of *E. scutalis* when controlling *O. afrasiaticus* in heavily infested varieties to acquire date fruits devoid of acaricide residues and dust mites.

## Simple Summary

This study demonstrates that the predatory mite *Euseius scutalis* can successfully develop and reproduce on the date palm mite *Oligonychus afrasiaticus* and exhibits strong population growth under laboratory conditions. Field releases of 400–600 individuals per tree effectively reduced *O. afrasiaticus* populations below the economic threshold in commercial date palm orchards. These findings support *E. scutalis* as a practical and sustainable biological control agent for managing date palm mite infestations.

## 1. Introduction

The Qassim region of Saudi Arabia is a principal center of date palm cultivation, playing a vital role in the Kingdom’s food security and agricultural export sector. The region produces more than 400,000 tons of dates annually, approximately 36% of Saudi Arabia’s total production. With over 11 million palm trees, Qassim cultivates more than 50 date varieties, including premium cultivars such as Barhi, Sukkari, Medjool, and Sagai. These high-quality dates are exported to more than 100 countries, underscoring global confidence in Saudi agricultural products [1]. However, this vital sector is continually threatened by numerous pests that reduce both the quality and quantity of the yield [2]. Among these, the date palm mite *Oligonychus afrasiaticus* (McGregor) (Acari: Tetranychidae) is a major pest of date fruits across the Middle East and North Africa, including Saudi Arabia, where it causes substantial losses by attacking green, developing dates [3,4]. Its infestations lead to discoloration, cracking, and significant market losses, making it a key constraint to sustainable date production in the region [5]. This mite thrives in hot, dry conditions and rapidly multiplies during the fruit-setting season, making early monitoring and integrated control essential [6].

In Saudi Arabia, control of date palm mite *O. afrasiaticus* has traditionally relied heavily on synthetic pesticides, with growers frequently spraying during the early growth stages and fruit setting. While these chemicals may provide short-term control, their intensive use has raised concerns about human health, contamination of soil and water, effects on non-target organisms, emergence of pesticide resistance, and environmental safety [7,8,9,10]. Given these concerns, it is increasingly important to adopt strategies that reduce dependence on synthetic pesticides, particularly in date palm production systems. One promising approach is the use of biological control agents, which offer an environmentally safe and economically viable alternative for managing phytophagous mites. As a core element of integrated pest management, biocontrol has demonstrated exceptional sustainability, reduced chemical inputs, mitigated resistance development, and supported ecosystem stability [11,12].

In date palm ecosystems, especially in regions such as Qassim, biological control offers a promising and increasingly important strategy for suppressing major pests, including *O. afrasiaticus*, supporting both crop productivity and environmental sustainability [5]. Most phytoseiid mites are generalist predators capable of developing and reproducing on a variety of prey when available. However, they can also survive for extended periods on a broad range of alternative foods such as pollen, fungal spores, nectar, honeydew, and plant exudates when prey is absent or scarce [13]. This dietary flexibility allows them to persist in agroecosystems, maintain baseline populations, and rapidly exploit pest outbreaks when they occur. Recent studies in Saudi Arabia, including those conducted in the Qassim region, highlight the need for more sustainable approaches, such as predatory mites, which have shown promising efficacy under laboratory conditions [5].

The predatory mite *Euseius scutalis* (Athias-Henriot) (Acari: Phytoseiidae) is one of the most important predatory mites used in biocontrol across warm regions, including Saudi Arabia, thanks to its ability to thrive at high temperatures and feed on many mite species, moth eggs, whitefly eggs, scale insects, thrips, and pollen [14,15,16,17,18,19,20]. Accordingly, the purpose of this study was to assess the impact of biocontrol of *O. afrasiaticus* using the predatory mite *E. scutalis* under lab conditions. Additionally, the study evaluated the effects of three release rates of the predatory mite *E. scutalis* (200, 400, and 600 individuals per tree) on the population density of the date palm mite *O. afrasiaticus* infesting the fruits of two date palm cultivars, Barhi and Sokary, in a commercial orchard located in the Qassim region of Saudi Arabia during the 2025 season.

## 2. Materials and Methods

### 2.1. Laboratory Experiments

Laboratory experiments were conducted in the Acarology Laboratory, Department of Plant Protection, Qassim University, Saudi Arabia. The development, survival, predation rate, fecundity, and life table parameters of *E. scutalis* feeding on *O. afrasiaticus* were evaluated at 32 ± 1 °C, 55 ± 5% RH, and a 14L:10D photoperiod. Colonies of *E. scutalis* were collected from unsprayed date palm orchards in the Al-Mulida district, Qassim region (26.297875° N, 43.790684° E), Saudi Arabia, and were adapted and propagated on kidney bean (*Phaseolus vulgaris* L.) leaves infested with spider mite *Tetranychus urticae* Koch (Acari: Tetranychidae) for at least 3 months in an incubator at 32 °C, 55% RH, and a 14L:10D photoperiod as a stock colony. *O. afrasiaticus* was obtained from heavily infested date palm trees from the same orchard. Colonies of predatory mites were maintained separately on rearing units made of kidney bean (*Phaseolus vulgaris* L.) leaves (3 cm diameter), which were positioned abaxial side up on cotton kept saturated with water, placed in Petri dishes (12 cm in diameter × 2.5 cm high), and maintained in an incubator at 32 ± 1 °C, 55 ± 5% RH, and a 14L:10D photoperiod. The predatory mites were transferred to fresh rearing units every 5–7 days. Distilled water was added every day to the Petri dishes to maintain a constant water level and preserve leaf freshness. The edges of bean leaves were covered with strips of damp tissue to avoid cross-contamination among colonies and prevent mite escape. The predators were continuously supplied with a mixture of mobile stages of date palm mite, *O. afrasiaticus*.

### 2.2. The Laboratory Consumption-Rate Trial

The laboratory consumption rate of *E. scutalis* feeding on *O. afrasiaticus* was evaluated at 32 ± 1 °C, 55 ± 5% RH, and a 14L:10D photoperiod. Eggs of the predatory mite were collected from the stock colony and individually placed in new rearing units to create age-synchronized cohorts for further experiments. A total of 30 eggs were randomly selected and individually transferred to new rearing units using a fine hairbrush. Different numbers of *O. afrasiaticus* were provided to the various developmental stages of *E. scutalis*. Each larva received 10 prey individuals of mixed mobile stages; each protonymph received 15; each deutonymph received 20; and each adult pair (female and male) was supplied with 50 prey. After 24 h, the number of prey consumed was recorded. To ensure successful multiple matings, each pair was kept together until both individuals died. To distinguish the consumption capacity of males from that of females, the predation rate of 30 males was evaluated under identical experimental circumstances. The number of prey consumed by males was then subtracted from the mean prey consumption of the adult pair, following the method of Moghadasi et al. (2014) [21]. For the sex ratio calculation, 30 eggs of the predator were collected and individually transferred to separate rearing units via a fine hairbrush. The larvae that resulted were reared to adulthood, after which each was mounted on a slide for the determination of sex.

### 2.3. Field Experiments

The present study was conducted during the fruiting season of 2025, extending from the beginning of May to the end of July in a date palm orchard located in Qassim University’s Agricultural Experiments Station in the Al-Mulida district (26.297875° N, 43.790684° E), Saudi Arabia. The orchard is characterized by an arid climate, low annual rainfall, and high summer temperatures typical of central Saudi Arabia. Standard agricultural practices used in the region were maintained during the study, with no acaricides applied throughout the trial period.

### 2.4. Date Palm Cultivars and Experimental Design

Two commercially important date palm cultivars, Barhi and Sukkari, were selected for evaluation. For each cultivar, 12 palm trees of similar age, height, vigor, and fruit load were chosen to minimize experimental variability. Four treatments were evaluated: three release rates of *E. scutalis* and an untreated control. The experiment was arranged as a Randomized Complete Block Design (RCBD) with three replicates (blocks). Within each block, the four treatments were randomly assigned to four experimental units (trees), resulting in three trees per treatment for each cultivar. Each palm tree was considered an experimental unit. Consequently, for each cultivar, nine trees received predator-release treatments (three trees per release rate), while three trees served as untreated controls.

### 2.5. Predatory Mite: Euseius scutalis

A laboratory-reared colony of the predatory mite *E. scutalis* was used. The colony was maintained on the two-spotted spider mite, *T. urticae*, as prey under controlled conditions (32 °C, 55% RH, and 14L:10D photoperiod). Predators were transported to the field in airtight containers and released early in the morning to avoid heat stress. Three release rates were tested: 200, 400, and 600 individuals/tree.

### 2.6. Target Pest: Oligonychus afrasiaticus

The date palm mite, *O. afrasiaticus*, naturally infested the orchard. No artificial infestation was performed. The experiment assessed the suppressive effect of predator releases on natural *O. afrasiaticus* populations present on the fruits. The experimental evaluation was conducted under heavy infestation conditions, with a mean density of approximately 52 mites per fruit in the Barhi cultivar and 36 mites per fruit in the Sokary cultivar.

### 2.7. Mite Release Procedure

Predatory mites were gently released into fruit clusters using soft camel hairbrushes. Release was performed once at the beginning of the trial, on the first of May, to ensure distribution around the fruit clusters. Each release rate was applied to three trees per cultivar, totaling nine treated trees per cultivar. Control trees received no predatory mites.

### 2.8. Fruit Sampling, Population Assessment, and Laboratory Examination

Before the release of the predator, and to determine the initial population of *O. afrasiaticus*, date fruits representing all bunches were randomly collected from each tree before treatment. The fruits were transported to the laboratory, where they were examined under a stereomicroscope, and the number of *O. afrasiaticus* mobile stages was recorded. Sampling was conducted weekly for twelve consecutive weeks during the 2025 season. Predatory mites were released on 1 May 2025, and the first post-release sample was collected seven days later, on 7 May 2025. Subsequent samples were collected at weekly intervals (14 May, 21 May, 28 May, and so forth) until a total of twelve sampling dates had been completed. From each tree, ten clusters were randomly picked. Five fruits per cluster were collected, totaling 50 fruits per tree per sampling date. Fruits were placed in labeled paper bags and transported to the acarology laboratory. Fruits were examined under a stereomicroscope (SD30, Olympus, Hachioji, Japan). The number of *O. afrasiaticus* mobile stages (larvae, nymphs, and adults) was recorded. Predatory mites observed on fruits were also counted to monitor establishment.

### 2.9. Data Analysis

Mean date palm mite *O. afrasiaticus* population densities were calculated for each treatment and sampling date. The Henderson–Tilton formula (1955) [22] was used to correct for natural population fluctuations relative to control trees. Data were subjected to ANOVA, and treatment means were compared using Tukey’s HSD test at *p* ≤ 0.05. Statistical analyses were performed using SAS statistical software (Version 9.4) (SAS Institute, Cary, NC, USA, 2017) [23]. Life-table parameters were calculated following the method of Birch (1948) [24].

The intrinsic rate of increase ($r_{m}$) was estimated by solving the Euler–Lotka equation:

$$\sum_{x=0}^{\infty }e^{-r_{m}x}l_{x}m_{x}=1$$

where x = age (days), $l_{x}$ = age-specific survival rate, $m_{x}$ = age-specific fecundity, and $r_{m}$ = intrinsic rate of natural increase.

The finite rate of increase (λ) was calculated as

$$\lambda =e^{r_{m}}$$

The net reproductive rate ($R_{0}$) was calculated as

$$R_{0}=\sum_{x=0}^{\infty }l_{x}m_{x}$$

The mean generation time (T) was estimated as

$$T=\frac{\ln R_{0}}{r_{m}}$$

where $R_{0}$ represents the average number of female offspring produced per female during her lifetime, T is the mean time between the birth of parents and that of their offspring, and λ is the multiplication factor per unit time. The sex ratio of the predator was analyzed using a Chi-square test.

## 3. Results

### 3.1. Development and Survival

The predatory mite, *E. scutalis*, completed its development on the date palm mite, *O. afrasiaticus,* under laboratory conditions (32 ± 1 °C, 50% RH, and 14:10 h L:D). All immature stages were able to feed and develop on *O. afrasiaticus*, and the overall mortality rate of immature stages was below 5%, indicating high suitability of this prey as a food source. The developmental duration differed slightly between females and males. Females completed in an average of 5.64 days, whereas males required 5.17 days to reach adulthood. These values reflect a relatively rapid developmental rate for a phytoseiid predator at this temperature.

### 3.2. Adult Longevity and Reproduction

The pre-oviposition, oviposition, and post-oviposition periods of the females average 1.73, 17.40, and 3.25 days, respectively. Adult female longevity averaged 10.36 days. During this period, females exhibited consistent oviposition activity. The mean daily oviposition rate was 2.81 eggs per female, resulting in a total fecundity of 48.90 eggs per female over the reproductive lifespan. The sex ratio of the progeny was strongly female-biased (0.70 female), a characteristic that enhances the population growth potential of the predatory mite, *E. scutalis* (Table 1).

### 3.3. Predation of E. scutalis on O. afrasiaticus 

The laboratory predation rate of *E. scutalis* feeding on *O. afrasiaticus* was evaluated at 32 ± 1 °C, 55 ± 5% RH, and a 14L:10D photoperiod. The larvae of *E. scutalis* were active immediately after hatching and began feeding immediately. Predation rates increased steadily with the predator’s developmental progression. Adults consumed substantially more prey than the nymphal stages, reflecting the higher energetic demands of mobility and reproduction. Immature females consumed an average of 28.90 individuals of *O. afrasiaticus* during their development. Among adult females, the highest daily consumption occurred during the oviposition period, during which each female consumed an average of 23.71 prey per day. Following this peak, daily predation gradually declined with advancing age. Over the entire life span, the predatory mite, *E. scutalis* females, exhibited the greatest cumulative predation capacity, consuming a total of 517.92 *O. afrasiaticus* individuals (Table 2).

### 3.4. Life Table Parameters

Life table parameters indicate that the predatory mite, *E. scutalis*, possesses a strong capacity for population increase when preying on the date palm mite, *O. afrasiaticus.* The intrinsic rate of natural increase (*r_m_*) was 0.281 females/female/day, suggesting strong potential for rapid population growth under warm conditions typical of date palm orchards. Consistently, the finite rate of increase (*λ*) reached 1.67, indicating that the population nearly doubled each day. The net reproductive rate (*R_o_*) was 11.80 females/female, confirming that each female more than replaced herself many times over during her lifetime. The mean generation time (*T_o_*) was 9.47 days, representing the combined duration of development and reproduction (Table 3). Collectively, these demographic parameters highlight the predator’s high reproductive efficiency and its strong potential for rapid establishment and population buildup when utilizing *O. afrasiaticus* as prey. The combination of short developmental time, low immature mortality, high fecundity, and a favorable sex ratio demonstrates that *O. afrasiaticus* is a suitable prey for the predatory mite, *E. scutalis.* The strong life table parameters further support the predator’s potential as an effective biological control agent against *O. afrasiaticus* in integrated pest management strategies.

### 3.5. Effect of Releasing Euseius scutalis on the Reduction in Oligonychus afrasiaticus on Two Date Palm Cultivars

Results in Table 4 show the effect of releasing three rates (200, 400, and 600 individuals) of the predatory mite *E. scutslis* on the reduction percentage of the date palm mite *O. afrasiaticus* infesting Barhi and Sokary date fruits. A clear dose-dependent trend was observed, where increasing predator release rates resulted in significantly higher reduction percentages in both cultivars. In the Barhi cultivar, the mean reduction percentages over the three months were 28.08, 55.56, and 71.08 9% for the release rates of 200, 400, and 60 predators, respectively (Table 4). Significant differences in pest reduction were observed among the three predator release rates, indicating that the quantity of predators released influenced the level of pest suppression. However, these differences do not necessarily imply a positive density-dependent response to increasing predator density. Similarly, in the Sokary cultivar, the corresponding reduction percentages were 23.48%, 49.03%, and 65.10%. Statistical analysis also showed significant differences among release rates (*p* ˂ 0.01).

Although the pattern of increasing reduction with higher predator release was consistent with Barhi, the absolute reduction values were significantly lower. Analysis of variance (ANOVA) revealed that the effect of predator release rate on the reduction percentage of *O. afrasiaticus* was highly significant in both date palm cultivars (*p* < 0.01), confirming a strong positive response to increasing predator density. Across all release rates, Barhi consistently showed significantly higher reduction percentages than Sokary, as confirmed by Tukey’s HSD test comparisons between cultivars at each release level.

Overall, these results confirm that the predatory mite, *E. scutalis*, is an effective biological control agent against *O. afrasiaticus*, particularly in cultivars with higher infestation potential, such as Barhi, and that its efficacy increases significantly with higher release rates. The findings also highlight the importance of cultivar-specific conditions, particularly initial pest density, in shaping the performance of predatory mites in date palm orchards. The results also highlight the importance of cultivar-specific conditions, particularly initial pest density, in shaping the performance of predatory mites in date palm orchards.

## 4. Discussion

The current study showed that the predatory mite *E. scutalis* can grow, survive, and reproduce when feeding on *O. afrasiaticus* in high-temperature lab conditions. The short development time, low mortality in immature stages, and high reproduction rate suggest that *O. afrasiaticus* is good prey for *E. scutalis*. This finding supports the predator’s potential role in controlling populations of this important date palm pest. The developmental times recorded for *E. scutalis* were 5.64 days for females and 5.17 days for males. These times are consistent with those reported for this predator when it preys on other tetranychid mites. Previous studies indicated that *E. scutalis* matures in about 5 to 7 days at temperatures between 25 and 32 °C when feeding on *Panonychus citri* (McGregor) [15], *Tetranychus turkestani* Ugarov [25], *Tetranychus urticae* Koch, and *Eutetranychus orientalis* Klein [18]. The low death rate among the young (5%) observed in this study supports this conclusion and matches mortality rates seen in other phytoseiids, such as *Amblyseius swirskii* Athias-Henriot [26] and *Phytoseius plumifer* (Canestrini and Fanzago), when they feed on suitable prey [20]. The results of the current study demonstrated that the oviposition rate was 2.81 eggs per female per day and the total fecundity was 48.90 eggs per female when the predatory mite, *E. scutalis*, fed on *O. afrasiaticus*, which was higher than that stated by Zergani et al. (2023) [25] (31.14 eggs/female) when feeding on *T. turkestani* and Nikolskii. Additionally, *E. scutalis* has demonstrated a total fecundity of 31.14, 39.7, 37.06, 30.66, 24.30, and 40.90 eggs/female on *T. turkestani* and Nikolskii, *P. citri*, *Tetranychus urticae* Koch, *Bemisia tabaci*, *Ricinus communis* (pollen), and *Colomerus vitis* (Pagenstecher) [15,17,18,20,25,27]. On the other hand, the findings of Bounfour and McMurtry (1987) for this predator feeding on *Tetranychus pacificus* McGregor eggs (59 eggs/female) were significantly higher than the value calculated in the present study [28]. When compared with other predatory mites commonly used in biocontrol, such as *Neoseiulus barkeri* Hughes and *Amblyseius swirskii*, the fecundity of *E. scutalis* in this study is also high. This reinforces its appropriateness for controlling *O. afrasiaticus* in warm climates [26]. The life table parameters acquired in this study further highlight the predator’s potential effectiveness. The rates of population growth were promising for *E. scutalis* fed on *O. afrasiaticus*. This was demonstrated by (*r_m_*), which was 0.281 female/female/day. The reported intrinsic rate of increase for *E. scutalis* on *T. turkestani* (0.253 at 25 °C) [25], *C. vitis* (0.211 at 33 °C) [26], *T. urticae* (0.191 at 26 °C) [27], *Aceria ficus* (Cotte) (0.218 at 28 °C), *E. orientalis* (Klein) (0.257 at 28 °C), and *Rhyncaphytoptus ficifoliae* Keifer (0.215 at 28 °C) [17] was lower than that acquired in this investigation when *E. scutalis* fed on *O. afrasiaticus*. Kasap and Şekeroğlu (2004) estimated an *r_m_* of 0.295 for *E. scutalis* fed on *Panonychus citri* at 30 °C [15]. Also, Shishehbor et al. (2022) estimated an *r_m_* of 0.330 for *E. scutalis* fed on *T. turkestani* at 25 °C, which is somewhat higher than our estimate [29]. The net reproductive rate (*R*_0_ = 11.80 females/female), finite rate of increase (λ) (1.67), and mean generation time (*T*_0_ = 9.47 days) observed here also align well with previously reported values for *E. scutalis*, indicating that *O. afrasiaticus* supports strong population growth. The strongly female-biased sex ratio (0.70 females) documented in this study is another advantage for biocontrol, as it increases the predator’s ability to control rapid population growth. Female-biased sex ratios are common among phytoseiids when feeding on high-quality prey [26], and the value observed here is comparable to those reported for *E. scutalis* on *T. turkestani*, *A. ficus*, *R. ficifoliae*, and *E. orientalis* [17,29].

The present study demonstrates the strong potential of *E. scutalis* as an efficient biocontrol agent against *O. afrasiaticus* on date palm trees, with predator release rate emerging as a key determinant of repression effectiveness. At higher release rates (200, 400, and 600 individuals), the percentage decrease increased significantly, confirming that *E. scutalis* responds favorably to more prey. In this study, the consistently greater reduction percentages in the Barhi cultivar compared with the Sokary cultivar indicate the importance of specific circumstances for predator performance. The Barhi cultivar typically has more initial infestations of *O. afrasiaticus*; this may help predatory mites establish and feed more efficiently by providing a consistent food source. Previous studies support this theory, indicating that Phytoseiid predatory mites frequently do better at moderate to high prey densities [30]. This environment allows for ongoing predation and quicker predator reproduction [31]. However, the lower reduction percentages in the Sokary cultivar could suggest lower initial *O. afrasiaticus* densities or habitat characteristics that hinder predator search efficiency or their capability to remain on the fruit surface. The release of *E. scutalis* led to a significant reduction in *O. afrasiaticus* populations throughout the experimental period compared with untreated control plots. This suppression was most pronounced during the middle and late seasons, when predator populations expanded rapidly and exerted intense predation pressure on *O. afrasiaticus*. The results of this study are consistent with earlier studies demonstrating the high predatory capacity and ecological adaptability of this species in orchard systems, where it effectively suppresses populations of tetranychid mites such as *T. urticae*, *E. orientalis*, and *O. afrasiaticus*, especially under hot and dry conditions where several other predators perform poorly [18]. Consistent with our findings, Zergani et al. (2023) stated that *E. scutalis* exhibits rapid development and high reproductive rates when feeding on spider mites or pollen, enabling it to establish quickly and sustain steady populations under field conditions [25]. Our results also support previous studies that the ecological success of *E. scutalis* is closely associated with its capacity to use substitute foods available in the orchard, such as date palm pollen, fungal spores, and natural plant exudates, enabling it to persist even when pest populations are low [29,32]. This characteristic is especially beneficial in arid areas, where prey abundance fluctuates seasonally, and environmental stressors can reduce the effectiveness of other predatory mites. Likewise, temperature tolerance seems to be another important factor underlying the effectiveness of *E. scutalis* in our system. Prior research has demonstrated that this predator maintains high reproductive potential and short developmental times at elevated temperatures, outperforming many other phytoseiid predators under heat stress [15,20]. The strong suppression of *O. afrasiaticus* observed in the warmest months of the year in our study enhances the suitability of *E. scutalis* for application in desert and semi-arid agroecosystems. This is particularly relevant for date palm orchards in Saudi Arabia, where summertime temperatures frequently surpass the thermal limitations of other predatory mites [26,32,33,34,35]. Our findings support these conclusions and highlight the potential of this predator as a core component of integrated pest management programs in orchard ecosystems, particularly in arid regions such as central Saudi Arabia, where the environmental circumstances favor its performance. The ability of *E. scutalis* to reproduce and establish within orchard ecosystems suggests that a single release may achieve pest suppression, provided that alternative food resources and favorable environmental conditions support the long-term persistence of predator populations. However, the long-term population dynamics of the predator were not assessed in the present study. Therefore, the optimal frequency of releases remains uncertain and is likely to vary according to orchard conditions, predator establishment, and pest density. Regular monitoring of both pest and predator populations is recommended to determine whether supplementary releases are required to maintain effective control of *O. afrasiaticus*. Future research should compare single and repeated release programs over multiple seasons to define long-term, cost-effective biological control strategies. Effective management of *O. afrasiaticus* should be based on an integrated pest management (IPM) approach that combines ecological, cultural, biological, and chemical tactics. Ecological and cultural components, including orchard sanitation, optimized irrigation practices, and habitat management, can help reduce pest outbreaks and improve the efficacy of natural enemies. These measures can be integrated with biological control agents and monitoring-based interventions to maintain mite populations below economic thresholds, thereby reducing reliance on chemical acaricides and supporting sustainable date palm production. Furthermore, the practical application of economic thresholds for *O. afrasiaticus* management requires regular and systematic monitoring of mite populations throughout the growing season. Attention should be given to identifying the timing of the initial migration of mites to fruit bunches, as this event may vary among date palm cultivars and environmental conditions. Accurate detection of early infestations would allow interventions to be implemented when populations approach the economic threshold, thereby maximizing control efficacy and minimizing unnecessary treatments. Therefore, integrating economic thresholds with cultivar-specific monitoring programs represents an essential component of sustainable IPM strategies for *O. afrasiaticus*.

## 5. Conclusions

The biological and demographic characteristics of *E. scutalis* observed in this study demonstrate that the predator is well adapted to feeding on *O. afrasiaticus*. Its rapid development, high fecundity, favorable sex ratio, and strong intrinsic rate of increase suggest that it can establish and proliferate effectively in date palm orchards, particularly under the high-temperature conditions typical of arid regions such as the Arabian Peninsula. These findings support the potential use of *E. scutalis* as a promising biological control agent within integrated pest management programs targeting *O. afrasiaticus*. In addition, the releases of *E. scutalis* can provide effective and sustained suppression of *O. afrasiaticus* populations in orchard ecosystems under arid environmental conditions. The results confirm the suitability of *E. scutalis* as a core biological control agent in integrated pest management programs, particularly in regions where fluctuating prey densities limit the performance of other natural enemies. By reducing *O. afrasiaticus* densities to below economic thresholds, the predator can minimize the need for chemical acaricides, thereby lowering production costs and reducing the risk of resistance development.

## Figures and Tables

**Table 1:** Life-history parameters (mean duration in days ± SE), survival, and reproductive rate of the predatory mite *Euseius scutalis* fed on date palm mite *Oligonychus afrasiaticus* at 32 ± 1 °C, 50% RH, and 14:10 h L:D.

Parameters	Female	Male	Survival to Adulthood (% ±SE)
Egg	2.10 ± 0.08	2.00 ± 0.12	98.51 ± 2.87
Larva	1.10 ± 0.12	1.05 ± 0.06	95.80 ± 3.26
Protonymph	1.20 ± 0.10	1.00 ± 0.08	95.41 ± 3.94
Deutonymph	1.24 ± 0.09	1.12 ± 0.11	95.00 ± 2.45
Pre-adult development time	5.64 ± 0.36	5.17 ± 0.28	
Pre-oviposition period	1.73 ± 0.20		
Oviposition period	17.40 ± 1.15		
Post-oviposition period	3.25 ± 0.35		
Total (longevity)	22.38 ± 1.55	20.24 ± 1.59	
Total lifespan	28.02 ± 1.47	25.41 ± 1.30	
Daily Fecundity	2.81 ± 0.16		
Total Fecundity	48.90 ± 2.07		

**Table 2:** Predation rate by different stages of the predatory mite *Euseius scutalis* fed on date palm mite *Oligonychus afrasiaticus* at 32 ± 1 °C, 50% RH, and 14:10 h L:D.

Predatory Stage	Sex	*Euseius scutalis*	
		No. of Attacked Mite Individuals	
		Total Average Mean ± SD	Daily Rate Mean ± SD
Larva	Female	4.68 ± 0.41	4.25 ± 0.22
	Male	4.00 ± 0.19	3.80 ± 0.28
Protonymph	Female	8.54 ± 0.42	7.11 ± 0.35
	Male	8.40 ± 0.44	8.40 ± 0.41
Deutonymph	Female	15.68 ± 0.57	12.64 ± 0.53
	Male	13.15 ± 0.29	11.74 ± 0.49
Total	Female	28.90 ± 1.14	8.16 ± 0.74
	Male	25.55 ± 0.98	8.05 ± 0.64
Pre-oviposition	Female	36.75 ± 1.22	21.24 ± 1.18
Oviposition	Female	412.60 ± 3.46	23.71 ± 1.24
Post-oviposition	Female	39.67 ± 0.63	12.20 ± 0.50
Longevity	Female	491.02 ± 4.58	21.94 ± 1.07
	Male	348.37 ± 2.94	17.21 ± 1.10
Life span	Female	517.92 ±4.82	18.48 ± 1.20
	Male	373.92 ±3.34	14.71 ± 0.95

**Table 3:** Life table parameters of the predatory mite *Euseius scutalis* fed on date palm mite *Oligonychus afrasiaticus* at 32 ± 1 °C, 50% RH, and 14:10 h L:D.

Parameters	*Euseius scutalis*
Net reproductive rate (*R_o_*)	11.80 ± 0.94
Mean generation time (*T_o_*)	9.47 ± 0.68
Intrinsic rate of increase (*r_m_*)	0.281 ± 0.04
Finite rate of increase (λ)	1.67 ± 0.03
Sex ratio (Female/total)	21/30

**Table 4:** Reduction (%) in the population of the date palm mite *O. afrasiaticus* after releasing the predatory mite *E. scutalis* on Barhi and Sokary date palm cultivars during three months in 2025.

Cultivars	Release Rates	Reduction Rate % in Date Palm Mite, *O. afrasiaticus* Population												
		Inspection Numbers												
		1	2	3	4	5	6	7	8	9	10	11	12	Mean
Barhi	200	5.24	9.16	12.37	15.33	19.85	25.73	29.49	37.12	42.74	44.31	47.69	48.00	28.08 a
	400	12.10	17.20	25.12	37.51	44.60	59.26	66.38	76.42	81.23	80.56	82.63	83.73	55.56 b
	600	28.30	37.38	46.59	59.82	67.19	84.20	86.54	86.60	88.11	89.25	89.40	89.60	71.09 c
Sokary	200	3.90	6.50	10.45	12.32	15.00	20.14	22.34	30.10	35.26	40.45	42.18	43.12	23.48 a
	400	7.25	13.28	25.42	34.15	40.36	53.26	59.16	70.34	70.58	71.00	71.65	71.93	49.03 b
	600	22.41	33.89	40.26	52.48	61.25	78.23	79.74	80.46	81.00	82.12	84.10	85.30	65.10 c

Different letters in each column denote significant differences (*p* < 0.01).

## Data Availability

The original contributions presented in this study are included in the article. Further inquiries can be directed to the corresponding authors.
